# Discrimination of human and animal bloodstains using hyperspectral imaging

**DOI:** 10.1007/s12024-023-00689-0

**Published:** 2023-09-18

**Authors:** Gary Sean Cooney, Hannes Köhler, Claire Chalopin, Carsten Babian

**Affiliations:** 1 Innovation Center Computer Assisted Surgery (ICCAS), Leipzig University, Leipzig, Germany; 2 Institute for Legal Medicine, Leipzig University, Leipzig, Germany

**Keywords:** Animal blood, Hyperspectral imaging (HSI), Support vector machine (SVM), Neighbourhood component feature selection (NCFS), Forensics

## Abstract

Blood is the most encountered type of biological evidence in violent crimes and contains pertinent information to a forensic investigation. The false presumption that blood encountered at a crime scene is human may not be realised until after costly and sample-consuming tests are performed. To address the question of blood origin, the novel application of visible-near infrared hyperspectral imaging (HSI) is used for the detection and discrimination of human and animal bloodstains. The HSI system used is a portable, non-contact, non-destructive method for the determination of blood origin. A support vector machine (SVM) binary classifier was trained for the discrimination of bloodstains of human (*n* = 20) and five animal species: pig (*n* = 20), mouse (*n* = 16), rat (*n* = 5), rabbit (*n* = 5), and cow (*n* = 20). On an independent test set, the SVM model achieved accuracy, precision, sensitivity, and specificity values of 96, 97, 95, and 96%, respectively. Segmented images of bloodstains aged over a period of two months were produced, allowing for the clear visualisation of the discrimination of human and animal bloodstains. The inclusion of such a system in a forensic investigation workflow not only removes ambiguity surrounding blood origin, but can potentially be used in tandem with HSI bloodstain age determination methods for rapid on-scene forensic analysis.

## Introduction

Blood is one of the most readily examined substances of the human body, playing a key role in investigative forensic science. A single drop of blood contains valuable information, such as its DNA, chemical composition, and morphology of associated bloodstains on surfaces. This information, along with bloodstain pattern analysis, can be utilised to reconstruct the events of a violent crime [1].

Basic characteristics of blood found at a crime scene are determined using serological presumptive tests, such as the Kastle-Meyer [2] and luminol tests [3]. These are fast, cheap, and efficient tests used in the discrimination of suspected blood from other substances of similar appearance. Luminol can detect highly diluted blood samples (10,000-fold) due to the efficient catalysation of chemiluminescence by the iron present in haemoglobin. Despite its sensitivity, luminol is limited by its non-specific reaction to other oxidants such as bleaches, or certain food such as horseradish. Problems with the interpretation of bloodstain patterns can occur if injured or killed animals (e.g. pets or wild animals) are also present at the crime scene [4]. Tests that use monoclonal anti-human Hb antibodies are available for the detection of the human origin of blood (e.g. Hexagon OBTI^®^ [5]); however, corresponding tests for the differentiation of different animal blood are lacking. The main drawback to these preliminary tests is their destructive nature [6]. In addition, immunoassays are prone to false positives, as certain animal haemoglobin is very similar to human haemoglobin.

Advanced analytical methods have become increasingly predominant in forensic sciences. Mass spectrometry and chromatography techniques have become commonplace in toxicology labs, and spectroscopic methods such as Raman spectroscopy, UV–vis spectroscopy, and Fourier transform infrared spectroscopy (FT-IR) have proven themselves in the analysis of blood [7–10]. These methods are often less destructive, or non-destructive, with less sample preparation when compared to presumptive tests [6]. Reflectance spectroscopy has the benefits of being a non-destructive method, with modern portable spectrometers enabling fast on-scene analysis. Nevertheless, reflectance spectroscopy is limited by long initial analysis times, which includes the identification and validation of characteristic sample biomarkers observed in a given sample’s reflectance spectrum. Furthermore, there is a high level of interpretation required to build classification and regression models for the respective identification and age estimation of a forensic sample for use outside of the laboratory environment.

In this paper, the need for a cheap and fast method to differentiate blood of unknown origin is addressed by the use of the non-contact modality hyperspectral imaging (HSI), coupled with chemometric methods to build a classification model that successfully discriminates human and animal bloodstains. HSI combines conventional imaging with spectroscopy, giving two-dimensions of spatial (*x*, *y*) and one dimension of spectral information (*λ*). The power of HSI lies in the ability to obtain a continuous spectrum for each pixel of a captured image. HSI has already been proven as an effective tool in various applications of forensic science including but not limited to airborne identification of unmarked graves [11, 12], detection of explosive residues [13], and for the non-contact analysis of forensic traces [14]. Concerning blood and bloodstain analysis by HSI, the determination of human bloodstain age is predominant [15–20]. While the determination of bloodstain age using HSI can objectively have greater value in a forensic investigation of a violent crime, the fact remains that the determination of blood origin is once again overlooked in the literature.

A controlled study using human blood and blood obtained from five common domestic animal species deposited onto white cotton was used as a preliminary investigation into the ability of visible-near infrared (vis–NIR) HSI to discriminate human and non-human bloodstains. Chemometric and machine learning methods were used to investigate and develop a binary human-animal classifier based on the reflectance HSI data. Image segmentation was performed on the captured colour images using a background detection algorithm in tandem with the trained classifier, effectively visualising the colour-coded discrimination of human versus animal bloodstains. The portable HSI system coupled to the human-animal classifier demonstrates the potential of this modality to significantly speed-up forensic investigation with on-site measurement capability.

## Materials and methods

### HSI system and acquisition

The commercial Specim IQ^®^ (Specim, Spectral Imaging Ltd., Oulu, Finland) was used for the capturing of hyperspectral images. This system features a push-broom scanner producing hypercubes in the range of 400–1000 nm with a spectral resolution of 7 nm (204 spectral bands, *λ*-axis). The number of effective pixels is 512 × 512 pix (*x*-, *y*-axis) and the camera fore optic provides a field of view of 31 × 31 degrees. Therefore, at a measurement distance of 30 cm between the camera and sample, a viewable area of 16.4 × 16.4 cm results in a theoretical maximum spatial resolution of 0.32 mm. Illumination was achieved using two tungsten-halogen broadband light sources (750 W each). The default recording mode with simultaneous white reference method, in which the white reference panel is measured alongside the target sample, was used for data acquisition. An integration time of 10 ms was used giving a recording time of 35 ms per hypercube. The reflectance data cube is calculated using the relation:

$${R}_{ij}\left(\lambda \right)=\frac{{RAW}_{ij}-{Dark}_{ij}}{{White}_{ij}}$$

where *R* is the reflectance, RAW is the raw data of light intensities measured, Dark is the instrument dark frame, which is the sensor baseline signal due to the camera electronics, White is the white reference plate intensity, and *i* and *j* are horizontal and vertical pixel indices, respectively. To reduce the interference, all external light sources including room lights were switched-off during image recording.

### Human and animal blood samples

Venous blood from 20 healthy human volunteers (10 male, 10 female, age 42 ± 16 years) was obtained from the Institute for Transfusion Medicine, University Hospital Leipzig. Only age and sex of the blood donor are known, with an identification number being used to pseudo-anonymise the donors. The 1.7 mL blood aliquots were collected into EDTA and refrigerated to avoid coagulation prior to measurement.

Blood from 20 pigs was obtained from Slaughterhouse Weiβenfels, Weiβenfels, in EDTA 1.7 mL aliquots. Venous blood from 20 cows was obtained from the Clinic for Hooved Animals, Faculty of Veterinary Medicine, Leipzig University in EDTA 1.7 mL aliquots. Cardiac blood from 16 mice (5 female CD1/CR, 5 male CD1, 6 female Sv129) was obtained from the Medical Experimental Centre III, University Hospital Leipzig, in EDTA 1.7 mL aliquots. Venous blood from 5 rats (3 female, 2 male SPRD) and venous blood from 5 rabbits (1 female, 1 male White New Zealand; 1 male, 2 female Chinchilla bastard) was obtained from the Medical Experimental Centre I, University Hospital Leipzig, in EDTA 1.7 mL aliquots.

All blood samples were transported to the Institute for Legal Medicine, University Leipzig, where the blood was then deposited onto white cotton fabric creating spots of ca. 5 cm^2^ which was let dry at room temperature for 10 min. Samples that were stored under refrigeration were allowed to warm to room temperature prior to probe preparation. The hyperspectral images were recorded using the SPECIM IQ^®^ camera under halogen light. The samples were left exposed under ambient conditions and recorded once daily for a week, and then intermittently up to 2 months. The data is summarised in Table 1 below.

**Table 1:** Unbalanced dataset of human and animals, including sample age range, number of recorded HSI images, ROIs, and total spectra

	**Sample No**	**Age [days]**	**HSI images**	**No. ROIs**	**Tot. spectra**
**Human**	20	0.1–32	21	103	231,075
**Animal**	66	0.1–49	59	250	454,450
Pig	20	0.1–42	8	83	180,000
Mouse	16	0.1–24	18	64	45,075
Rat	5	0.1–49	12	18	35,750
Rabbit	5	0.1–49	12	18	50,625
Cow	20	0.1–42	9	67	145,000

### Classification framework

**Fig. 1:**
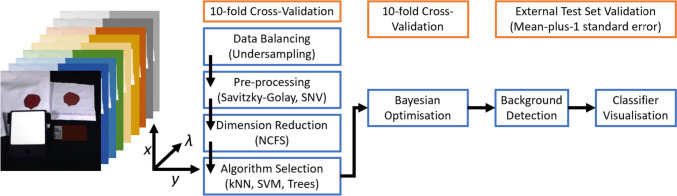
Classification framework for HSI data cubes of human and animal bloodstains

The aim of the classification was the automatic identification and segmentation of blood with respect to human and animal blood using HSI. The classification framework is summarised in Fig. 1 below. HSI data was annotated using GIMP (The GIMP development Team, 2019) to create regions of interest (ROI) of the blood samples consisting of approximately 25 × 25 pixels. Microsoft Excel (Microsoft Corporation 2019) was used for the documentation and analysis of results. Data balancing and data pre-processing were performed using custom scripts written in MATLAB (version 9.8; R2020a, The MathWorks Inc.), with machine learning algorithm selection and optimisation being performed using the Statistics and Machine Learning Toolbox™ and Imaging Processing Toolbox™, both being provided by MATLAB.

#### Data balancing and pre-processing

The species classes include different numbers of individuals and recorded HSI images which in turn lead to drastically different numbers of spectra per class. To build an unbiased classification model, the data was balanced using undersampling; that is, observations from the majority class were randomly excluded to decrease its size to be comparable to that of the minority class. In addition, to build a classification model that is independent of sample age, the data must be logically distributed to equally represent changes in the blood spectra with time. As the effect of time on blood composition is the most explored method in age determine of bloodstains [6, 17, 21], the effect of time on the absorption ratio of blood components is well documented. The ROIs were divided into bins that follow the natural exponential series *f(x)* = *e*^*x*^ where *x* = *0, 1, 2, 3* and *f(x)* = *1, 2.718, 7.389, 20.086*. This gives the time interval bins: 0.0–1.0, 1.0–3.0, 3.0–7.0, 7.0–20.0, and 20.0–55.0 days. This was in order to capture the exponential-like decrease in haemoglobin derivatives oxyhaemoglobin (HbO2), and increase in methaemoglobin (metHb) and haemachrome (HC) with respect to degradation over time [17]. Within each species class, the age group bins were first balanced using undersampling to that of the least represented bin. The animal classes were then balanced with respect to each other, so each species was equally represented, before balancing to the human class by the random exclusion of animal observations, maintaining age group distribution. Human observations were assigned the binary response 1, while all animal observations were assigned the binary response 0, thus forming the two classes.

The spectral window of 400–1000 nm (204 spectral bands) was initially truncated to 435–965 nm due to the low camera sensitivity and low power of the halogen light source beyond this range [18]. A Savitzky-Golay filter [22] with polynomial order of two and window length of nine spectral bands was implemented to smooth the reflectance spectra giving an effective wavelength range of 445–955 nm (169 spectral bands). Spectra were then normalised using the SNV transform [23], which auto-scales the data giving a mean reflectance of zero and standard deviation of one. The SNV reflectance spectra were then used in feature selection before the training of classification models.

#### Feature selection

Neighbourhood component feature selection (NCFS) is a neighbour-based feature weighting algorithm proposed by Yang et al. [24]. NCFS was implemented to reduce data dimensionality and identify regions of interest in the blood spectra that contribute to the successful discrimination of human and animal blood. The stochastic gradient descent (SGD) solver algorithm, with solver-batch size of 1000 observations, was used to estimate feature weights. The initial learning rates were turned with a subset size of 10,000 observations. The best value for the regularisation parameter λ that minimises the generalisation error is expected to be a multiple of the inverse of the number of observations *n*. Given the large number of observations in the training set (144,020 spectra), the expected value is λ = 6.943 × 10^−6^ and therefore can be approximated as zero. Without a regularisation parameter, all features have a weight greater than 0, and therefore, a feature weight threshold was implemented. Thresholds of 0.5, 0.55, 0.6, 0.65, 0.70, and 0.75 times the maximum feature weight were used to select 92, 68, 43, 34, 26, and 16 of the most important wavelengths as determined by the NCFS algorithm. These features were then used to train simple k-Nearest Neighbour classifiers (*k* = 1) of the training set. The 1-Nearest Neighbour algorithm was chosen for its simplicity, speed of training, and non-parametric nature, leading to the effective evaluation of best feature selection. The trained 1-Nearest Neighbour classifiers were evaluated based on their F1 scores. The F1 score is: ${F}_{1}=2\times \frac{precision \times recall}{precision + recall}$, and arguably captures the model’s performance better than the accuracy, recall, and precision values individually.

#### Background detection

**Fig. 2:**
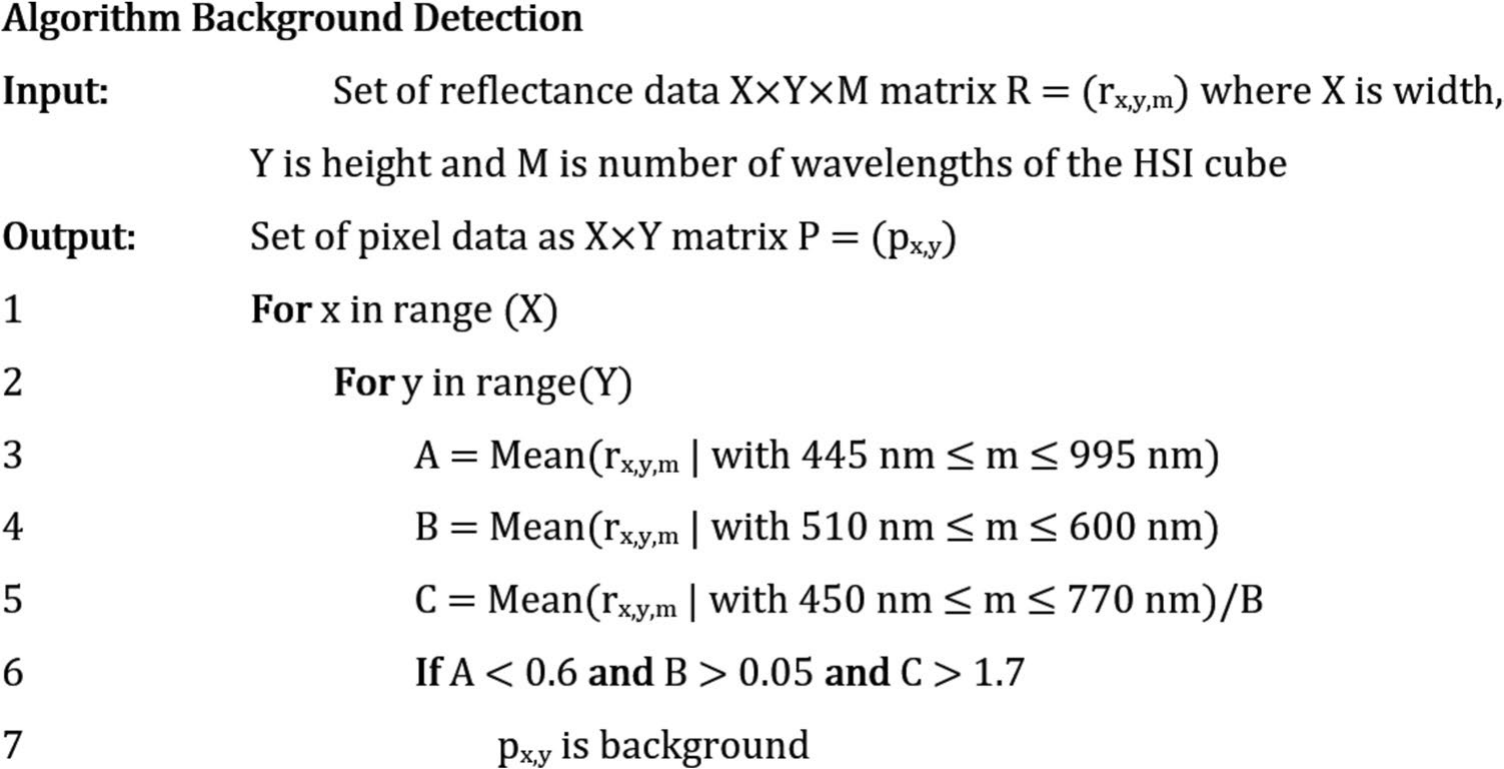
Modified algorithm background detection [25] (ABG) to detect background prior to classification

Prior to image classification, a decision tree algorithm for background detection was implemented. The definitions of the wavelength parameters A, B, and C of the algorithm background detection [25] (ABD, Fig. 2) were modified from their original application in biomedical tissue analysis to exclude all background information in the HSI images except for the bloodstains.

The algorithm parameters A, B, and C attempt to exclude spectra that have features that are not characteristic to blood and bloodstains. Parameter A removes constant high-valued reflectance spectra, such as the white cloth deposition surface. Parameter B is based on the Q-bands of haem, which are predominant low-reflectance in spectra of blood. Parameter C attempts to capture the proportionality between the low-reflectance region of the haem Q-bands and the high-reflectance far-red region of a typical blood reflectance spectrum. The parameter threshold values A < 0.6, B > 0.05, and C > 1.7 were empirically determined as the optimum values for background detection. The image morphological noise removal technique “closing” was performed on the ABD binary gradient mask.

#### Classification

The data was split into training and test sets as per Table 2. This was further randomly divided into 66% validation and 34% test set to be used in the validation of optimised models and the final chosen model, respectively.

**Table 2:** Training set and test set partition with balancing

	**No**	**Training set**	**Spectra**	**Test set**	**Spectra**
**Human**	20	15	72010	5	28125
**Animal**	66	53	72010	13	28125
Pig	20	16	14402	4	5625
Mouse	16	13	14402	3	5625
Rat	5	4	14402	1	5625
Rabbit	5	4	14402	1	5625
Cow	20	16	14402	4	5625

Five binary classification algorithms were initially tested using 10-fold cross-validation: SVM with polynomial and Gaussian kernels, decision tree, bagged tree, and k-NN. The models were assessed based on their F1 scores and area under the ROI curves (AUC). The SVM with polynomial kernel, bagged tree, and k-NN models were selected for further optimisation of the classifier model parameters. Bayesian optimisation was implemented, which attempts to minimise an objective function *f(x)* for *x* by using an acquisition function *a(x)* to determine the next hyperparameter point for evaluation. The acquisition function “expected-improvement-per-second-plus” was used to evaluate the goodness of fit [26, 27]. Thirty iterations were used to evaluate the models with Bayesian optimisation. For the SVM, the kernel scale and box constraint hyperparameters were simultaneously optimised. The distance metric and number of neighbours were optimised for the k-NN model, while the bagged decision tree was optimised based on number of learning cycles and number of leaves within the tree. The optimised models were then tested using the validation data and the F1 scores were compared. The polynomial SVM was selected for further optimisation, and the degree of overfitting estimated using the training error, 10-fold cross-validation error, and validation error of the 9 “best” iterations as determined by the Bayesian optimisation algorithm. The optimal SVM model was selected based on the mean-plus-1 standard error of the smallest mean cross-validation error.

## Results

### Feature selection

**Fig. 3:**
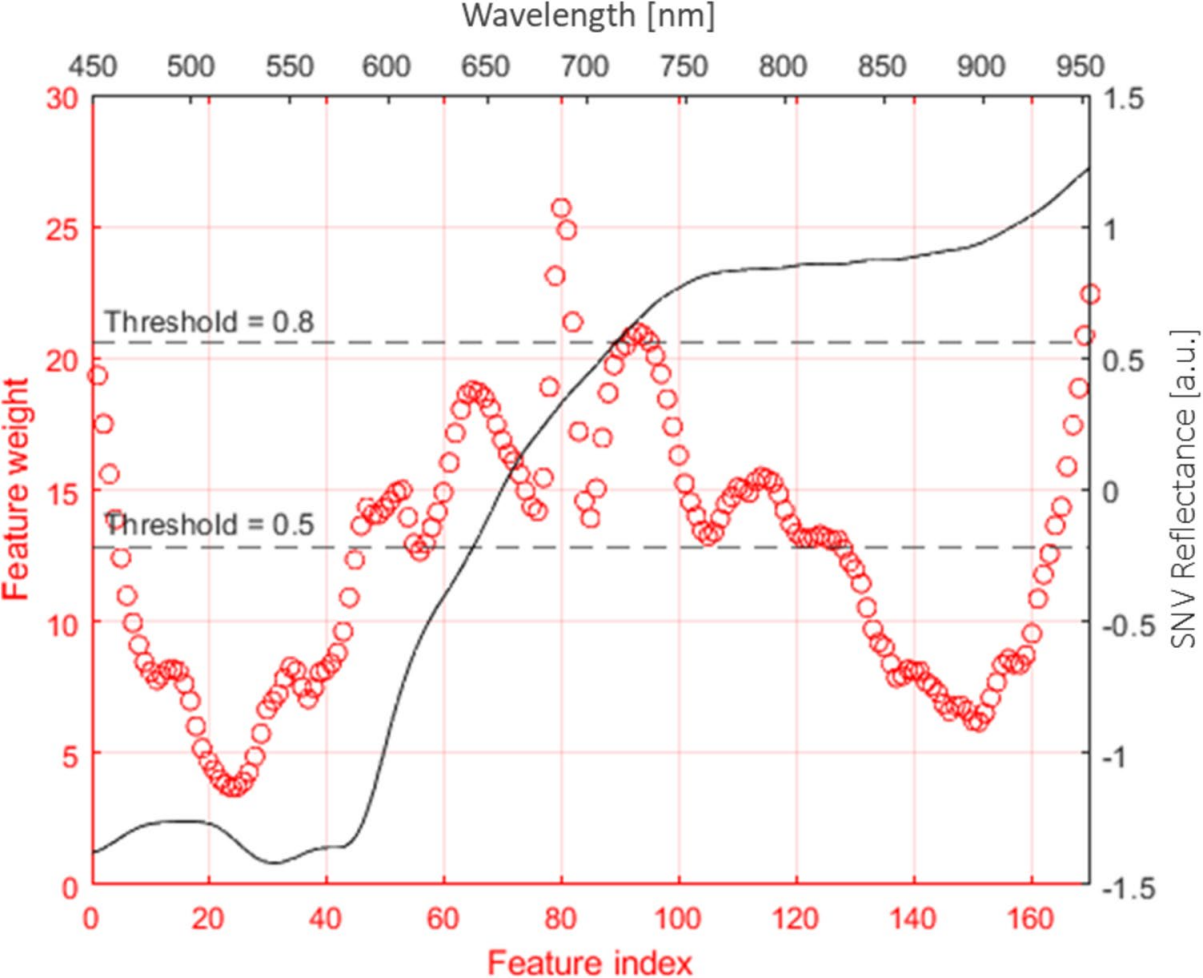
NCFS feature weights as a function of feature index (red circles) with threshold lines of 50% and 80% the maximum feature index weight. The average human blood spectrum is plotted as a secondary axis (black; x-axis wavelength [nm], y-axis SNV reflectance [a.u.]) to guide the eye

To identify spectral bands of greatest interest as determined by the NCFS algorithm in a typical blood spectrum, the average SNV reflectance human blood spectrum was plotted as a secondary axis (black) to the feature weights (red) (see Fig. 3). Threshold values of between 50 and 80%, in increments of 5% of the feature of maximum weight (feature 80, weight = 26.12), were used to select the most important wavelengths for use in further classification. Six k-NN models where *k* = 1 were trained using the threshold values and the model statistics compared to a k-NN classification model without feature selection (threshold = 0, 170 wavelengths). The effect of information loss with fewer features resulting in more misclassifications is reflected in the F1 scores presented in Table 3 below. Comparing to model 1 without feature selection, there is a 2.8% decrease in F1 score with model 3, which has a feature weight threshold of 0.55. Model 4 has a relative decrease of 2.3% compared to model 3. In addition, model 3 reduces the data dimensionality by 60% from 170 to 68 features, which is 15% greater than model 2 of 92 features. Therefore, the optimal threshold value of 0.55 with 68 features (wavelengths) was selected for use in further classification model development, as this is the best trade-off between data reduction and information retention.

**Table 3:** NCFS k = 1, k-NN model F1 scores to determine the optimal number of features based on the prediction of the validation set. Model no. 3 with threshold of 0.55 and 68 wavelengths was chosen as optimal number of features

**Model No.**	**Threshold**	**No. Features**	**F1 score**
1	0	170	0.9255
2	0.50	92	0.9125
**3**	**0.55**	**68**	**0.8971**
4	0.60	43	0.8740
5	0.65	34	0.8487
6	0.70	26	0.8463

### Bayesian optimisation

Bayesian optimisation was used to optimise hyperparameters of k-NN, bagged tree, and SVM models with 10-fold cross-validation. The acquisition function “expected-improvement-per-second-plus” was used to evaluate the next hyperparameter point for evaluation. The best observed feasible point of each of the k-NN, bagged tree, and SVM models as determined by the Bayesian optimisation are presented in Table 4 below.

**Table 4:** Results of first Bayesian optimisation for k-NN, bagged tree and 3rd-order polynomial SVM including optimised parameter values, the observed objective function value of 10-fold cross-validation, and corresponding F1 score

**Model**	**Parameters**		**Objective function**	**F1 score**
k-NN	No. neighbour Distance metric	5 Euclidean	0.0118	0.847
Bagged tree	Learning cycles	476	0.0143	0.807
3-poly SVM	Box constraint Kernel scale	88.7 1.83	**0.0047**	**0.862**

The 3rd-order polynomial SVM was determined as the best classification model for further evaluation. The further optimisation of the SVM was set to run 30 iterations but was prematurely terminated at 21 iterations for reaching a total objective function evaluation time of 150,000 s (41.67 h).

**Fig. 4:**
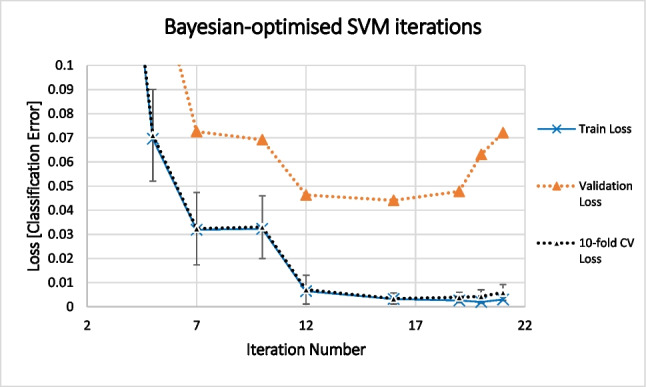
Comparison of training loss, CV-loss, and validation loss of Bayesian-optimised SVM iterations. Based on mean plus 1 standard error for the smallest mean cross-validation error, the parameters of iteration 16 are chosen as optimum for the SVM classification model

For the final SVM binary classification model, the parameters of box constraint = 74.185 and kernel scale = 2.7558 were used as per iteration 16 of the Bayesian optimisation (Fig. 4). The retrained model was tested with the independent test set (19,125 observations) and the model statistics calculated. Presented in Table 5 is the model statistics of the predicted test data. This model has an accuracy and F1 score above 95% in the discrimination of human and animal bloodstains up to 32 and 49 days, respectively.

**Table 5:** Model statistics of SVM binary classifier. *TOT* total number of observations predicted, *PREV* prevalence; a measure of class distribution, *ACC* accuracy, *PPV* positive predictive value or precision, *TPR* true positive rate or sensitivity, *TNR* true negative rate or specificity

**TOT**	**PREV**	**ACC**	**PPV**	**TPR**	**TNR**	**F1 score**
19125	0.4996	0.9564	0.9671	0.9470	0.9663	0.9569

### Image classification of bloodstains

**Fig. 5:**
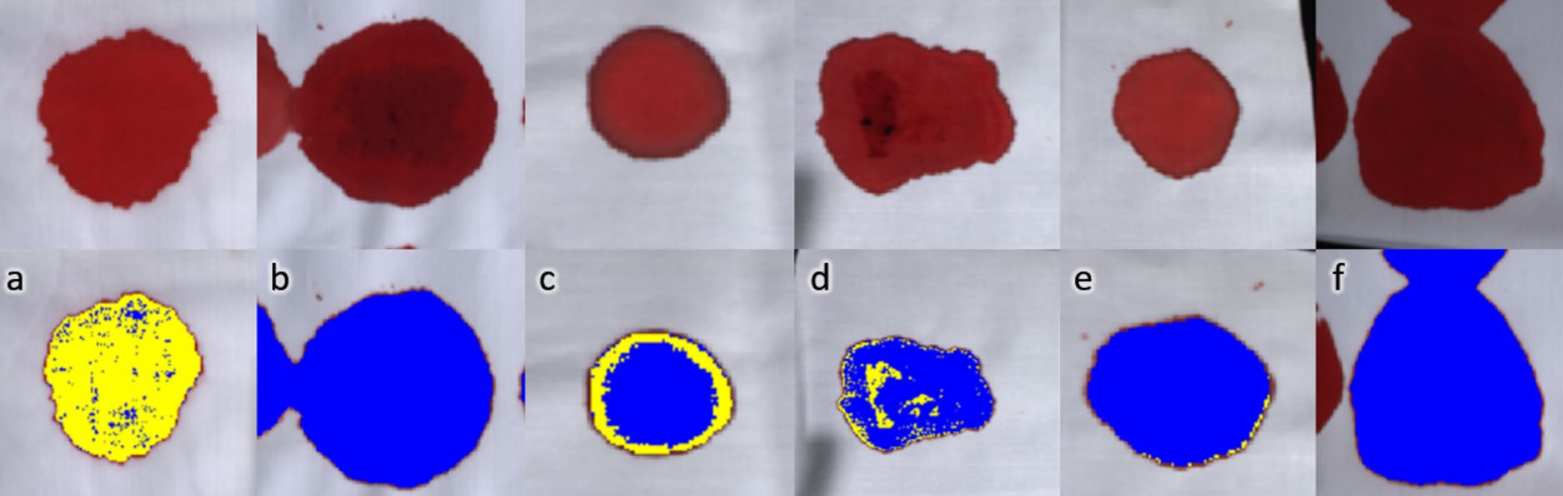
HSI colour images of fresh bloodstains (0.1 days; top row) and with SVM classification mask (bottom row). The sample species are **a** human, **b** pig, **c** mouse, **d** rat, **e** rabbit, and **f** cow. Yellow pixels are classified as “human” and blue as “animal”

**Fig. 6:**
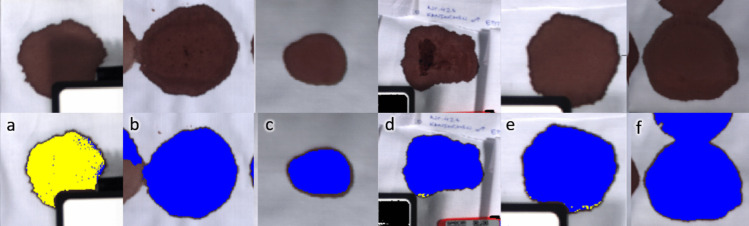
HSI colour images of aged bloodstains (5 weeks; top row) and with SVM classification mask (bottom row). The sample species are **a** human, **b** pig, **c** mouse, **d** rat, **e** rabbit, and **f** cow. Yellow pixels are classified as “human” and blue as “animal”

The HSI images associated with the test data set were processed and classified using the trained SVM binary classifier. One individual from each of the animal classes and one human sample is presented in Figs. 5 and 6 below. The colour images are presented in the first row followed by the classified images directly under. The presented images are taken from the classification bins to illustrate the model’s performance with respect to aging. Pixels that are classified as “human” by the SVM classifier are coloured with a yellow mask, while pixels classified as “animal” are coloured with a blue mask. No further image processing was applied after SVM classification.

## Discussion

As seen in Fig. 3, the red circles of NCFS feature values form a feature weight-index curve that illustrates the correlation between wavelengths and their respective importance in distinguishing human and animal blood reflectance spectra. It can be concluded that the wavelength bands of most importance (in descending order of feature weight) are centred around: 680, 955, 725, 445, 645, 775, and 600 nm. These peaks can then be used to infer the contributions of blood components to the observed reflectance spectra and give an indication of the potential underlying differences between human and animal blood. As blood spectra from all samples are included in the NCFS calculation, it can be assumed that the feature weights are independent of blood age and thus spectral differences are attributed to the varying composition. This is evident in the alpha and beta Q-bands at ca. 550 and 575 nm which are a result of oxy- and deoxyhaemoglobin derivatives [28], having feature weights below 50% of the maximum weighted feature by the NCFS algorithm. That being said, the region between 600 and 750 nm is often attributed to the derivatives HHb (broad peak 600–700, sharp peak at 760 nm) and MetHb at 630 nm.

In building the machine learning models, a train/test split method was implemented as model test validation. Ten-fold cross-validation was also implemented within the NCFS optimisation and validation of the Bayesian-optimised SVM iterations. The mean-plus-1 standard error was used in determining the optimal model which is the least likely to experience overfitting, and this model was tested on the independent test set. An alternative validation method to the train/test split validation is the leave-one-out cross-validation (LOOCV). Despite LOOCV being a very robust method for testing models, it is also computationally expensive. In addition, it can be assumed that the variation between individual spectra of a given bloodstain is less than that between different bloodstains. A modified form of LOOCV is the leave-one-patient-out cross-validation, where the data is divided into folds based on the number of individuals in the dataset and the complete data from each individual is used in turn as test set. Nevertheless, this approach would require significantly more calculation and would not be expected to improve the binary classification, considering the relatively small differences between 20 bloodstains (or less) per species.

The optimised SVM classifier has good statistical values for the independent test dataset. All major predictors including accuracy, precision, sensitivity, specificity, and F1 score are all above 95% (round up) as per Table 5. The model’s predictive power in the discrimination of human and animal blood is evident in the correctly classified images of Figs. 5 and 6. The bloodstains in general across all species are correctly classified, where the human sample is classified yellow signifying the “human” classification label, and pig, mouse, rat, rabbit, and cow blood are all classified blue as “animal”. SVMs perform well in blood discrimination, and are seemingly only out performed by more complex neural networks [29]. Książek et al. [30] reported that deep neural networks were found to have no clear advantage over SVMs when the training and test set came from the same hyperspectral image. However, a 9% increase in overall accuracy was obtained for the deep learning architectures when the training set and test set was derived from different images. This demonstrates the continued interest in exploring deep learning methods for effective blood discrimination in forensic science. Future extensions of this work could evaluate the performance of deep neural networks in comparison to established machine learning methods for the determination of blood origin.

The misclassification on the periphery of the bloodstain (Fig. 5c: mouse blood, day 0.1 and Fig. 5d: rat blood, day 0.1) is most likely the result of capillary flow [31], where the edges of the bloodstain appear darker due to the higher concentration of haemoglobin and its derivatives. This has been observed in bloodstain age studies that used cotton [32] as a deposition surface and due to haemolysis with improper handling [33]. Nevertheless, for the reflectance measurements in this study, the region of interest was centred in the middle of the bloodstain for each sample.

The parameters of the trained SVM could be used as a starting point for the new multi-species classifier, which could also include a broader animal dataset. The inclusion of more species such as cat, dog, horse, chicken, sheep, goat, fish, and reptiles into the animal repertoire would increase the confidence in the classifier’s ability to truly discriminate human blood from that of any animal. Variables, such as age, dilution, humidity, and temperature, should be further explored, as these could influence the degradation of bloodstains.

The deposition surface of white cotton was selected due to its high reflectivity, which aids the visualisation of bloodstains. It is spectroscopically featureless, meaning there was no contribution to the measured vis–NIR reflectance of blood. Following fabrics, materials such as wood, stone, ceramics, plastics, and metals need to be investigated. This would give conclusive insight into the classifiers’ ability to function in any given condition encountered at a crime scene. The open-source dataset for the evaluation of blood detection by Romaszewski et al. [34] could act as a good starting point for this purpose.

The developed classifier could be modified and/or coupled to a separate algorithm for the simultaneous determination of bloodstain age. In the reconstruction of a crime, one of the primary goals of the investigator is the determination of the time when a crime is committed. For these reasons, there has been significant interest in the research of deposited blood age [35]. Raman spectroscopy [36], IR spectroscopy [37, 38], and reflectance spectroscopy [32, 39] have all been used in blood age determination. In particular, vis–NIR HSI similar to that used in this work has been successfully used for the age estimation of bloodstains [14, 16, 17].

## Conclusions

The novel application of vis–NIR HSI was successfully used in the detection and discrimination of human and animal bloodstains on white cotton and achieved an F1 score of 95.7% when classifying the independent test dataset.

The featured HSI system has significant benefits in the discrimination of human and non-human bloodstains. Its portable, non-contact, and non-destructive nature lends itself to forensic investigation, having the capability of on-site detection and evaluation of forensic evidence. Thus, relevant human blood traces can be distinguished from animal blood traces at the crime scene and preferably secured for molecular genetic investigations. This, in turn, reduces time and money that would be otherwise lost to lengthy laboratory analysis procedures. Such a system could be expanded to include blood age-determination methods as previously outlined and has the potential to become the workhorse of forensic investigation.

## Key points

1. The recognition and classification of blood traces at crime scenes is an important part of forensic case work.
2. The distinction between human and animal blood has so far only been possible through sample-consuming laboratory tests.
3. Hyperspectral analysis enables the contact-free recognition and differentiation of such blood traces.
4. The portable SPECIM IQ hyperspectral camera is suitable for use at the crime scene and evaluates the traces directly on the display.

## Data Availability

The datasets generated during and/or analysed during the current study are available from the corresponding author on reasonable request.

## Code availability

The code generated during the current study is available from the corresponding author on reasonable request.
